# Psychomotor speed as a predictor of functional status in older chronic heart failure (CHF) patients attending cardiac rehabilitation

**DOI:** 10.1371/journal.pone.0235570

**Published:** 2020-07-02

**Authors:** Antonia Pierobon, Nicolò Granata, Valeria Torlaschi, Chiara Vailati, Alice Radici, Roberto Maestri, Claudia Pavesi, Marinella Sommaruga, Lidia Gazzi, Giorgio Bertolotti, Simona Sarzi Braga, Mauro Monelli, Emanuela Zanelli, Anna Giardini

**Affiliations:** 1 Psychology Unit, Istituti Clinici Scientifici Maugeri, IRCCS, Montescano Institute, Pavia, Italy; 2 Department of Biomedical Engineering, Istituti Clinici Scientifici Maugeri, IRCCS, Montescano Institute, Pavia, Italy; 3 Department of Cardiac Rehabilitation, Istituti Clinici Scientifici Maugeri, IRCCS, Montescano Institute, Pavia, Italy; 4 Psychology Unit, Istituti Clinici Scientifici Maugeri, IRCCS, Camaldoli Institute, Milano, Italy; 5 Psychology Unit, Istituti Clinici Scientifici Maugeri, IRCCS, Lumezzane Institute, Brescia, Italy; 6 Psychology Unit, Istituti Clinici Scientifici Maugeri, IRCCS, Tradate Institute, Varese, Italy; 7 Department of Cardiac Rehabilitation, Istituti Clinici Scientifici Maugeri, IRCCS, Tradate Institute, Varese, Italy; 8 Subacute Care, Istituti Clinici Scientifici Maugeri, IRCCS, Camaldoli Institute, Milano, Italy; 9 Department of Cardiac Rehabilitation, Istituti Clinici Scientifici Maugeri, IRCCS, Lumezzane Institute, Brescia, Italy; Scuola Superiore Sant'Anna, ITALY

## Abstract

**Background:**

The association among psychological, neuropsychological dysfunctions and functional/clinical variables in Chronic Heart Failure (CHF) has been extensively addressed in literature. However, only a few studies investigated those associations in the older population.

**Purpose:**

To evaluate the psychological/neuropsychological profile of older CHF patients, to explore the interrelation with clinical/functional variables and to identify potential independent predictors of patients’ functional status.

**Methods:**

This study was conducted with a multi-center observational design. The following assessments were performed: anxiety (Hospital Anxiety and Depression Scale, HADS), depression (Geriatric Depression Scale, GDS), cognitive impairment (Addenbrooke’s Cognitive Examination Revised, ACE-R), executive functions (Frontal Assessment Battery, FAB), constructive abilities (Clock Drawing Test, CDT), psychomotor speed and alternated attention (Trail Making Test, TMT-A/B), functional status (6-minute walking test, 6MWT) and clinical variables (New York Heart Association, NYHA; Brain Natriuretic Peptide, BNP; left ventricular ejection fraction, LVEF; left ventricular end diastolic diameter, LVEDD; left ventricular end diastolic volume, LVEDV; tricuspid annular plane systolic excursion, TAPSE).

**Results:**

100 CHF patients (mean age: 74.9±7.1 years; mean LVEF: 36.1±13.4) were included in the study. Anxious and depressive symptoms were observed in 16% and 24,5% of patients, respectively. Age was related to TMT-A and CDT (r = 0.49, p<0.001 and r = -0.32, p = 0.001, respectively), Log-BNP was related to ACE-R-Fluency subtest, (r = -0.22, p = 0.034), and 6MWT was related to ACE-R-Memory subtest and TMT-A (r = 0.24, p = 0.031 and r = -0.32, p = 0.005, respectively). Both anxiety and depression symptoms were related to ACE-R-Total score (r = -0.25, p = 0.013 and r = -0.32, p = 0.002, respectively) and depressive symptoms were related to CDT (r = -0.23, p = 0.024). At multiple regression analysis, Log-BNP and TMT-A were significant and independent predictors of functional status: worse findings on Log-BNP and TMT-A were associated with shorter distance walked at the 6MWT.

**Conclusions:**

Psychological and neuropsychological screening, along with the assessment of psychomotor speed (TMT-A), may provide useful information for older CHF patients undergoing cardiac rehabilitation.

## Introduction

Chronic heart failure (CHF) affects more than 37 million people in the world and represents one of the diseases with the highest impact on health outcomes. It is a chronic condition associated with high rates of hospitalization, re-admissions, severe disability and high risk of mortality. In developed countries, 1–2% of the adult population suffers from this chronic condition and the rates are even higher, reaching 10%, in people aged 70 years or over [1].

Although many studies have focused on specific somatic symptoms (dyspnea, fatigue and physical exertion), mental health aspects (affective/mood disorders) and cognitive function/impairment, few have investigated the inter-relationship between these factors [2]. Anxiety and depression are reported to be present in 20–30% of CHF patients [3]. The presence of depressive symptoms increases the rates of CHF appearance, especially in older patients and those with previous cardiovascular diseases [4,5]. Moreover, anxiety and depressive symptoms are associated with a worsening of primary outcomes in terms of frequent re-hospitalizations, increased healthcare costs and higher risk of mortality [5,6]. Since the effect of anxiety on self-care management can be masked by the stronger influence of depression on self-care management, it is important to consider the wide spectrum of mental comorbidities in patients with CHF [3,7], including cognitive impairment. Indeed, in a recent systematic review and meta-analysis, the odds ratio for cognitive impairment in the CHF population in case-control studies was 1.67 [95% confidence interval (CI) 1.15–2.42] and the prevalence of cognitive impairment in CHF cohorts (n = 26 studies, 4176 participants) was 43% (95% CI 30–55) [8]. The presence of cognitive impairment has a great impact on CHF patients’ health status as it contributes to low self-care, poor adherence to clinical prescriptions, increased re-hospitalizations and higher risk of mortality [9–11]. In particular, poorer global cognitive score and working memory, psychomotor speed, and executive function dysfunctions are significant predictors of mortality [12].

Psychological and neuropsychological dysfunctions and their association with functional and cardiac variables have been extensively reported, but these investigations often consider both younger and older CHF patients without distinguishing them [12–14]. Hence, in older (age 65+ years) and oldest-old (80+) CHF patients, these associations deserve to be specifically investigated [15].

Cardiac rehabilitation is a recommended and suitable treatment for CHF patients since it reduces symptoms, decreases disability, increases participation in physical and social activities and improves functional outcomes [16,17]. Psychological and neuropsychological aspects, functional status and clinical variables can be all considered and measured in a cardiac rehabilitation setting [18].

The aims of this multi-center observational study were to evaluate the psychological and neuropsychological profile of older CHF patients undergoing inpatient rehabilitation and to investigate the relationships between neuropsychological and psychological data with clinical and functional ones. Furthermore, a specific investigation was performed to explore which of the considered variables might be independent predictors of functional status.

## Materials and methods

### Participants

All CHF patients aged over 65 years consecutively admitted to the Maugeri Clinical Scientific Institutes IRCCS Cardiac Rehabilitation Departments of Montescano, Camaldoli, Tradate and Lumezzane, Italy, for inpatient cardiac rehabilitation between January and December 2017, were screened for admission. The first part of the recruitment was performed at the Montescano Institute (first six months of the year), followed by Camaldoli, Tradate and Lumezzane Institutes (two months for each Institute).

CHF was defined as: I) signs (e.g. elevated jugular venous pressure, pulmonary crackles and peripheral edema) and symptoms of HF [New York Heart Association (NYHA) functional class II-IV] in the presence of reduced ejection fraction (LVEF <40%); or II) signs and symptoms of HF (e.g. elevated brain natriuretic peptides and significant structural heart disease/diastolic dysfunction) with preserved (LVEF ≥50) or mid-range ejection fraction (LVEF 40–49%) [1].

The exclusion criteria were: severe clinical conditions (chronic inflammatory diseases, severe and acute respiratory diseases, neoplastic diseases, cerebrovascular diseases), no Italian education, severe visuo-perceptive deficits, low subjective motivation/interest or refusal to undergo the evaluation, severe psychiatric disorders (at medical psychiatric evaluation) and severe cognitive deterioration, evaluated through the Mini-Mental State Examination (MMSE) (MMSE score ≤18.3) [19] that is included in the Addenbrooke’s Cognitive Examination–Revised (ACE-R) [20,21].

### Materials

#### Anxiety and depression

Psychological assessment was performed with the Geriatric Depression Scale (GDS) [22] for depressive symptoms and the Anxiety subscale of the Hospital Anxiety and Depression Scale (HADS-A) [23] for anxiety symptoms. The 30-point GDS is a questionnaire specifically aimed at assessing depression in old patients, while HADS-A consists of seven specific questions to evaluate anxiety for hospitalized patients. Depressive symptoms were classified as absent for a GDS score 0–10, mild (GDS score 11–15), moderate (GDS score 16–20), or severe (GDS score 21–30). Anxiety symptoms were classified as absent (HADS-A score 0–7), mild (HADS-A score 8–10), moderate (HADS-A score 11–14), or severe (HADS-A score 15–21).

#### Cognitive screening

Neuropsychological assessment consisted of cognitive screening and specific executive function tests. The MMSE [19] is a brief 30-point questionnaire that we used to exclude patients with severe cognitive impairment (score ≤18.3). Scores were adjusted for age and education according to the Magni et al. distribution [19]. This MMSE is included in the ACE-R, [20,21] which is a screening test designed for the early detection of cognitive impairment. ACE-R is considered to be useful for screening degenerative dementias when shorter tests (i.e. MMSE) are less sensitive [12] and its sub-scales can be useful to screen impairments in specific areas of cognitive functioning. It is divided into five sub-scales that analyze five cognitive domains: Attention-Orientation, Memory, Verbal Fluency, Language, and Visuospatial abilities [20].

#### Executive and verbal functions

For the executive functions screening, the Frontal Assessment Battery (FAB) was administered [24]. It is a neuropsychological test composed of six sub-tests that explore specific cognitive or behavioral domains related to the frontal lobes such as conceptualization, mental flexibility, motor programming, sensitivity to interference, inhibitory control and environmental autonomy. Specific tests were added to more deeply evaluate other cognitive functions. Both language and executive functions were evaluated with the phonemic fluency test [25], in which subjects were asked to list in the space of one minute all the words that they know starting with the letters "F"—"A"—"S". The semantic fluency test (similar modality but applied to the specific categories of cars, fruits and animals) was administered to evaluate the semantic cognitive residual resources [26]. The Clock Drawing Test (CDT) was administered only in the free-drawn condition with a score range 0–15 [27]. A correct execution of the CDT requires not only intact perceptual abilities but also a correct functioning of the constructive abilities, of verbal comprehension, knowledge and understanding of the numerical system, and abstract thinking. We used the Trail-Making Test (TMT parts A and B) [28] to assess psychomotor speed and alternated attention. In part A, the subject has to connect, in the proper order, twenty-five encircled numbers randomly arranged on a page. Part A requires the use of visual search processes and assesses psychomotor speed. In Part B, the subject has to draw a line to connect alternating numbers and letters, starting with number 1 and letter A, in rising sequence. Part B requires switching/cognitive flexibility and assesses alternated attention. Differently from the other tests in our battery, TMT scores represent the time spent to complete the task; therefore, the higher the scores, the worse is the performance.

The scores of all the aforementioned neuropsychological tests were adjusted for age, sex, and educational level. All the tests have good psychometric properties, are validated in the Italian population, and normative data are available [20,24–28].

#### Clinical and functional variables

New York Heart Association (NYHA), Brain Natriuretic Peptide (BNP), left ventricular ejection fraction (LVEF), left ventricular end diastolic diameter (LVEDD), left ventricular end diastolic volume (LVEDV), tricuspid annular plane systolic excursion (TAPSE) are clinical variables considered to describe CHF sample. LVEF and Log-BNP were also considered in statistical analysis for their clinical predictive value [1].

The 6-minute walking test (6MWT) is considered an adequate submaximal test and is commonly used to measure the functional exercise capacity in individuals with CHF. It is a self-paced exercise test that entails measurement of distance walked over a span of 6 minutes, that is better tolerated and more reflective of daily activities than other maximal exercise tests [17].

### Data collection

All patients were admitted to an inpatient rehabilitation program comprehensive of medical history and physical examination, evaluation of laboratory parameters, ECG, chest X-ray, color-Doppler echocardiogram, medical therapy optimization, exercise testing (6 minutes walking test, 6MWT), educational sessions, exercise training (cycle-ergometer and/or treadmill, leg ergometer, breathing exercises), psychological counselling, and metabolic evaluation with a personalized diet when needed.

The patients signed an informed consent for all procedures and explanations concerning the study. The study was approved by the Institutional Review Board and Central Ethics Committee of the ICS Maugeri SpA SB (CEC) (approval number: CEC N.927, 27/06/2013).

During the first week of admission, all enrolled patients filled in a socio-demographic form and then underwent psychological and neuropsychological assessment. The psychological and neuropsychological research assessment was performed in a dedicated room of the Psychology Unit of each Institute and divided in two sessions: 1) ACE-R, HADS and GDS and 2) FAB, CDT, TMT-A/B, semantic and word fluency that were administered the subsequent day in order to avoid an interference effect. Trained psychologists evaluated the patients according to standardized administration and scoring procedures. The patients were supported throughout the testing period (30’ each session) to maintain motivation and to elicit the optimal level of performance; a break was always allowed if necessary. Furthermore, patients underwent a clinical psychological treatment: they received the results of their research assessment and they underwent a psychological interview according to the rehabilitation protocol and the specific needs of the patient.

### Statistical analysis

A sample size of 67 patients was deemed to be necessary to disclose, with a power equal to 80% and a Type I error of 0.05, a difference between an expected Pearson correlation coefficient equal to 0.3 and a correlation coefficient equal to 0 assessing the relationship between cognitive screening (ACE-R, FAB, CDT, TMT, word and semantic fluency), psychological (HADS-A, GDS) and clinical/functional variables (log BNP, LVEF, age, 6MWT). We anticipated that during the study period (the year 2017) we would be able to enroll many more patients among all those hospitalized in the ICS Maugeri cardiac rehabilitative department.

Descriptive statistics are reported as mean ± standard deviation (SD) for continuous variables and as number (percentage) for discrete variables.

The association between pairs of variables (age, illness duration, Log-BNP, LVEF, 6MWT and ACE-R total score and subtests, FAB, TMT-A/B, CDT, word fluency and semantic fluency adjusted scores) was assessed by Pearson correlation coefficient r. The associations between functional status as assessed by the 6MWT and clinical (LVEF, Log-BNP), demographic (age, gender), neuropsychological (ACE-R Memory, ACE-R Fluency, FAB and TMT-A adjusted scores) and psychosocial variables (HADS-A and GDS) was assessed by multiple regression analysis. Multicollinearity was checked computing the variance inflation factor (VIF). Variables showing VIF value greater than 10 were considered potentially problematic.

All statistical tests were two-tailed and statistical significance was set at p<0.05. All analyses were carried out using the SAS/STAT statistical package, release 9.2 (SAS Institute Inc., Cary, NC, USA).

## Results

In this multi-center cross-sectional observational study, 123 CHF patients aged ≥ 65 years, in NYHA class II-IV, consecutively admitted for inpatient rehabilitation, were screened for inclusion. Of these, 23 patients were excluded for the following reasons: clinical exacerbation during hospitalization (n = 3), no Italian education (n = 3), visuo-perceptive deficits (n = 3), low subjective motivation, or refusal, to undergo the evaluation (n = 7), severe psychiatric diseases (n = 1), and severe cognitive impairment (MMSE≤18.3) (n = 6). The final study population consisted of 100 patients.

Table 1 reports the demographic, psychosocial, functional and clinical characteristics of the study sample. Moderate/severe anxiety symptoms were present in 16% of patients, and moderate/severe depressive symptoms in 24.5% of patients.

**Table 1:** Psychosocial, demographic, clinical and functional characteristics of the study sample (n = 100).

	n(%)
**Gender**	
Male	74 (74.0)
Female	26 (26.0)
**Education (school years)**	
<5	43 (43.0)
6–8	29 (29.0)
9–13	25 (25.0)
>14	3 (3.0)
**Lives alone**	
No	76 (76.0)
Yes	24 (24.0)
**Marital status**	
Married/common-law partner	54(54.0)
Widower	23(23.0)
Unmarried	15 (15.0)
Separated/divorced	8 (8.0)
**Current occupation**	
Retired	92 (92.0)
Employed	8 (8.0)
**Primary caregiver**	
Nobody	19 (19.0)
Husband/wife/partner	53 (53.0)
Daughter/son	16 (16.0)
Other family member/carer	12 (12.0)
**Smoker**	
No	4 (4.0)
Yes	30 (30.0)
Ex-smoker	66 (66.0)
**NYHA class**	
I-II	27 (27.0)
III-IV	73 (73.0)
**Comorbidities**	
Diabetes	31 (31.0)
Hypertension	60 (60.0)
Hypercholesterolemia	54 (54.0)
**Anxiety symptoms (HADS-A)**	
None (0–7)	65 (65.0)
Mild (8–10)	19 (19.0)
Moderate (11–14)	13 (13.0)
Severe (15–21)	3 (3.0)
**Depressive symptoms (GDS) (n = 98)**	
None (0–10)	58 (58.9)
Mild (11–15)	15 (14.7)
Moderate (16–20)	17 (16.7)
Severe (21–30)	8 (7.8)
**Clinical and functional characteristics**	**M (SD)**
Age (years)	74.9 (7.1)
Illness duration (months)	164.8 (142.4)
Log_BNP	7.8 (1.1)
LVEF (%)°	36.1 (13.4)
LVEDD (mm)	61.9 (12.5)
LVEDV (ml)	168.5 (81.7)
TAPSE (mm)	18.1 (4.5)
6MWT (meters)	287.6 (110.1)

° LVEF:reduced, 68 (68%); mid-range, 21 (21%); preserved, 11 (11%).

HADS-A, Hospital Anxiety and Depression Scale-Anxiety; GDS, Geriatric Depression Scale; NYHA, New York Heart Association; BNP, Brain Natriuretic Peptide; LVEF, left ventricular ejection fraction; LVEDD, left ventricular end diastolic diameter; LVEDV, left ventricular end diastolic volume; TAPSE, tricuspid annular plane systolic excursion; 6MWT, 6-minute walking test.

Table 2 shows the frequency distribution of the neuropsychological assessment results. For each neuropsychological test, the results were divided into impaired, borderline and normal scores. Few patients obtained impaired (4%) or borderline (11%) scores at cognitive screening (ACE-R), while higher percentages of impaired scores can be found in the executive functions (FAB) scores (56%), in the psychomotor speed (TMT-A) scores (24.2%) and in the alternated attention (TMT-B) scores (51.5%).

**Table 2:** CHF patients' neuropsychological assessment results (n = 100).

**Cognitive screening**	**n (%)**
**ACE-R**	** **
**ACE-R total (0–100)**	
Impaired scores (≤71.78)	4 (4.0)
Borderline scores (71.79–79.86)	11 (11.0)
Normal scores (>79.86)	85 (85.0)
**ACE-R Attention Orientation (0–18)**	
Impaired scores (≤14.73)	6 (6.0)
Borderline scores (14.74–16.34)	11 (11.0)
Normal scores (>16.34)	83 (83.0)
**ACE-R Memory (0–26)**	
Impaired scores (≤14.47)	8 (8.0)
Borderline scores (14.48–17.85)	15 (15.0)
Normal scores (>17.85)	77 (77.0)
**ACE-R Fluency (0–14)**	
Impaired scores (≤6.01)	16 (16.0)
Borderline scores (6.02–7.54)	9 (9.0)
Normal scores (>7.54)	75 (75.0)
**ACE-R Language (0–26)**	
Impaired scores (≤18.83)	0 (0.0)
Borderline scores (18.84–21.83)	7 (7.0)
Normal scores (>21.83)	93 (93.0)
**ACE-R Visuospatial (0–16)**	
Impaired scores (≤10.73)	3 (3.0)
Borderline scores (10.74–12.32)	9 (9.0)
Normal scores (>12.32)	88 (88.0)
**Executive and verbal functions**	**n (%)**
**FAB (0–18)**	
Impaired scores (≤13.4)	56 (56.0)
Borderline scores (13.5–14.3)	10 (10.0)
Normal scores (>14.3)	34 (34.0)
TMT-A (n = 99)*	
Impaired scores (≥94)	24 (24.2)
Borderline scores (93–69)	10 (10.1)
Normal scores (≤68)	65 (65.7)
TMT-B (n = 99)*	
Impaired scores (≥283)	51 (51.5)
Borderline scores (282–178)	4 (4.1)
Normal scores (≤177)	44 (44.4)
**CDT**	
Impaired scores (≤7.56)	9 (9)
Borderline scores (7.57–9.37)	6 (6)
Normal scores (≥9.38)	85 (85)
**Semantic Fluency**	** **
Impaired scores (≤24)	5 (5.0)
Borderline scores (25–29)	23 (23.0)
Normal scores (>29)	72 (72.0)
**Word Fluency**	** **
Impaired scores (≤17.4)	17 (17.0)
Borderline scores (17.5–21.3)	11 (11.0)
Normal scores (>21.3)	72 (72.0)

* TMT scores represent the time spent (in seconds) to complete the task: the higher the scores, the worse is the performance.

ACE-R, Addenbrooke’s Cognitive Examination-Revised; FAB, Frontal Assessment Battery; TMT, Trail Making test; CDT, Clock Drawing Test.

**Fig 1:**
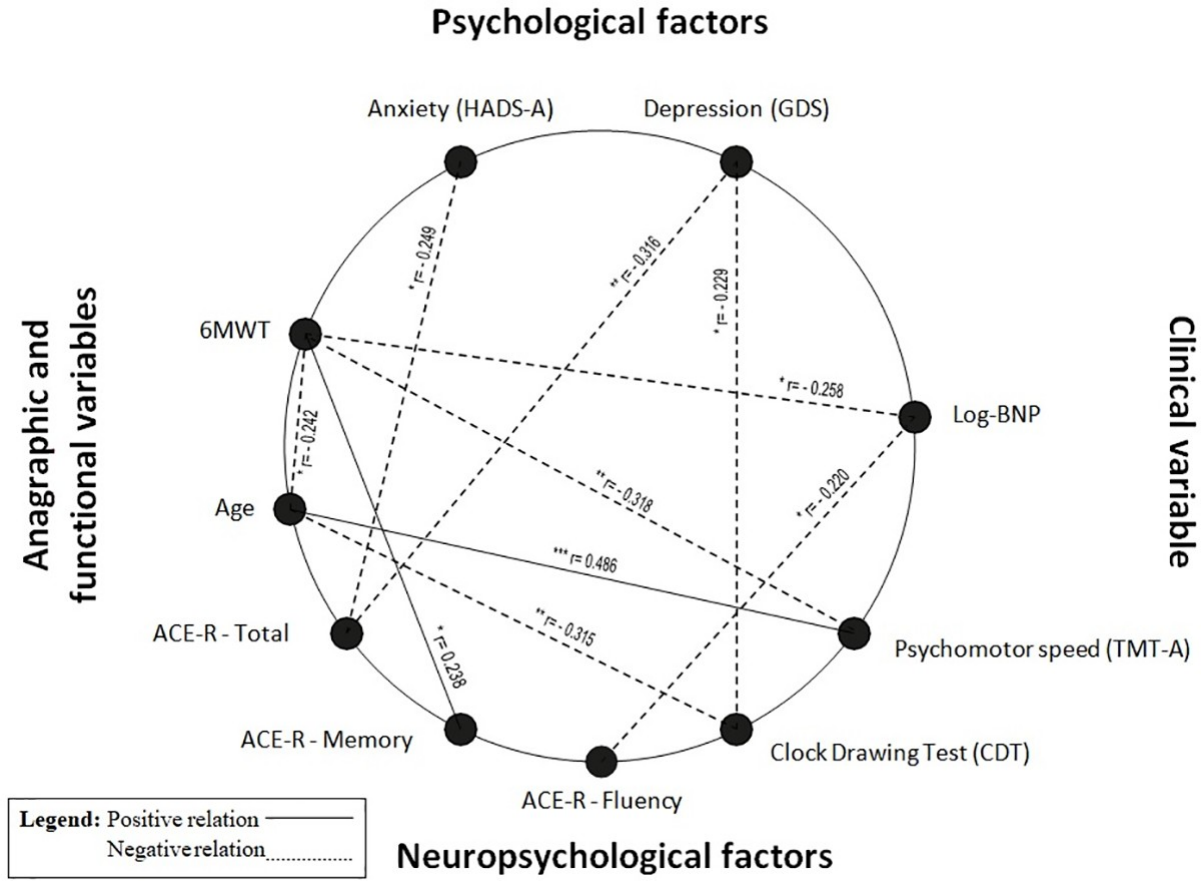
Positive or negative significant relations between demographic/functional/clinical variables and psychological/neuropsychological factors. P-value: ***<0.001, **<0.01, * <0.05; r value: correlation coefficient; HADS-A, Hospital Anxiety and Depression Scale-Anxiety; GDS, Geriatric Depression Scale; BNP, Brain Natriuretic Peptide; TMT-A, Trail Making Test–A; ACE-R, Addenbrooke’s Cognitive Examination-Revised; 6MWT, 6-minute walking test.

As to the significant association between demographic/clinical/functional variables and neuropsychological adjusted scores, age was related to TMT-A and CDT (r = 0.49, p<0.0001 and r = -0.32, p = 0.001, respectively), Log-BNP was related to ACE-R-Fluency, (r = -0.22, p = 0.034), and 6MWT was related to ACE-R-Memory and TMT-A (r = 0.24, p = 0.031 and r = -0.32, p = 0.005, respectively). Both anxiety and depression symptoms were related to ACE-R-Total (r = -0.25, p = 0.013 and r = -0.32, p = 0.002, respectively) and depressive symptoms were related to CDT (r = -0.23, p = 0.024) (Fig 1).

At multiple regression analysis, among the set of variables considered potential predictors of functional status as assessed by the 6MWT (age, gender, ACE-R-Memory, ACE-R-Fluency, FAB, TMT-A, depression, anxiety, Log-BNP and LVEF), Log-BNP and TMT-A were identified as significant and independent predictors of functional status (Table 3). Multicollinearity, as assessed by the variance inflation factor was not a problem, with values ranging from 1.33 for ACE-R-F to 1.74 for TMT-A. Holding all other variables constant, worse values for the clinical variable Log-BNP (parameter estimate -41.1, p = 0.0024) and a worse performance on the task of psychomotor speed, assessed by the TMT-A (parameter estimate -0.89, p = 0.025), were associated with shorter distance walked at the 6MWT.

**Table 3:** Multiple regression analysis results.

	Parameter Estimate	t Value	P value	95% Confidence Limits	
Age	0.61256	0.27	0.7888	-3.945	5.170
Gender	42.34875	1.33	0.1882	-21.319	106.016
ACE-R_Memory	3.54078	1.04	0.3040	-3.294	10.375
ACE-R_Fluency	-2.29482	-0.43	0.6706	-13.039	8.450
FAB	-4.40451	-0.99	0.3254	-13.293	4.484
HADS-A	41.33077	1.09	0.2812	-34.720	117.381
GDS	-33.84938	-0.98	0.3319	-103.092	35.393
LVEF	-1.53615	-1.27	0.2076	-3.949	0.877
Log_BNP	-41.09292	-3.18	0.0024	-66.987	-15.199
TMT-A	-0.89548	-2.29	0.0255	-1.677	-0.114

Model F value = 2.44, p = 0.016. Rsquare = 0.30.

ACE-R, Addenbrooke’s Cognitive Examination Revised; FAB, Frontal Assessment Battery; HADS-A, Hospital Anxiety and Depression Scale- Anxiety; GDS, Geriatric Depression Scale; LVEF, left ventricular ejection fraction; BNP, Brain Natriuretic Peptide; TMT-A, Trail Making Test (Part A).

## Discussion

Our study provides useful information concerning the psychological and cognitive screening in older and oldest-old CHF patients and an in-depth analysis of the cognitive impairments through a specific neuropsychological evaluation. In addition, it analyzed the relationship between cognitive performance and clinical, functional and psychological variables and it investigated which of the psychological, neuropsychological and clinical variables were independent predictors of the functional status.

Our psychological screening highlighted the presence of moderate/severe anxiety (16%) and depressive (24.5%) symptoms, in accordance with the existing data in the literature [3]. A brief psychological screening tool can help clinicians identify anxiety and depressive symptoms that might require specific counseling, also in older CHF patients. These data emphasize the need for a psychological intervention to treat emotional distress and support the process of adaptation related to suffering from a chronic disease [29].

For our neuropsychological assessment, we chose the ACE-R as a screening tool, because it enables a deeper analysis of global and specific cognitive functioning [30]. Particularly interesting were the data concerning the ACE-R subtests. In fact, the memory domain (including tasks of anterograde, retrograde memory, recall and recognition) and verbal fluency domain (phonological and semantic) showed a significant general decline due to deficits in executive functions and memory abilities, in line with the literature [31,32]. The impaired results of specific screening test for executive functions (56%) were in line with our findings from the ACE-R subtests: marked difficulties emerged that can be linked to procedural dysfunction, such as difficulty of conceptualization, mental flexibility, motor programming, sensitivity to interference and inhibitory control. Our findings on executive functions were further supported by the high percentage of impaired scores in psychomotor speed (24.2%), assessed through TMT-A and alternated attention (51.5%), assessed through TMT-B.

It is important to highlight that the well-known relations within neuropsychological, psychological and clinical variables were not considered because they were not the focus of the present study.

ACE-R total and CDT adjusted scores both have a negative relation with depression scores, meaning that patients with worse performance at neuropsychological tests presented a higher presence of depression symptoms. These data highlight that our sample’s results are in line with the existing literature about the prevalence of emotional distress in people with cognitive impairment [33,34]. In fact, affective and emotional dysregulation are common in preclinical and prodromal dementia syndromes, often revealing in advance neurodegenerative changes and a progressive cognitive decline [35]. Other relations frequently described in literature can be found between cognitive impairment and clinical variables [36] between psychological/neuropsychological factors and clinical variables [13] or between psychological/neuropsychological factors and functional variables [37,38].

Also in our study we found significant relations between ACE-R Fluency and Log-BNP and other interesting associations between 6MWT and both ACE-R Memory subtest and psychomotor speed (Fig 1). This link between the distance covered on the 6MWT, which decreases with age, could reflect the influence that a CHF condition can exert on attention, memory and motor efficiency of CHF patients, synthesizing a general cognitive-motor slowing [12,38]. This hypothesis finds further support in our regression model, where Log-BNP and psychomotor speed independently predicted distance covered at the 6MWT. This might indicate that clinically severe CHF patients with decreased psychomotor speed also have functional complications in the context of a general cognitive-motor slowing.

In a multidisciplinary and rehabilitative setting, this fact could be extremely challenging. Further research is needed to find a possible algorithm to predict patients’ functional status, starting from both clinical status and the result of psychomotor speed evaluation (through a test, the TMT-A, that can be administered also at the patients’ bedside) in older CHF patients who cannot undergo a 6MWT evaluation. Moreover, this statement acquires greater importance considering the significant impact that psychomotor speed has on CHF patients’ self-care [39].

An accurate psychological and neuropsychological screening is time saving and it requires a brief specific training. This kind of screening could play a pivotal role as it allows to implement an effective multicomponent and tailored rehabilitative intervention [18,29,40,41].

### Strengths and limitations

Considering the strengths, it is a multi-center study, providing the first Italian ACE-R data on older and oldest old CHF patients. Moreover, it considers many different variables and their relationships by comparing psychological and neuropsychological aspects to clinical and functional ones and it provides interesting cues for future research on the results concerning psychomotor speed and functional status. As to limitations, the neuropsychological in-depth evaluation focused only on executive and attentional aspects, whereas a wide spectrum evaluation could be useful to identify the possible presence of other impaired cognitive domains in older CHF patients. Secondly, because of our small sample size, our conclusions may not be generalized to all CHF patients, in particular to CHF outpatients.

## Conclusions

Based on our findings, more attention should be paid to the link between cognitive and emotional variables, functional status and CHF clinical data. Concerning the psychosocial aspects, a more in-depth psychological interview is recommended to further investigate the evidence from the screening tests in a wider psychosocial perspective and to support patients who have emotional distress.

Consistent with our findings, besides cognitive screening, an evaluation of psychomotor speed could be useful to further investigate and to predict CHF older patients’ functional status when they are unable to perform the dedicated functional test (6MWT).

In conclusion, a psychological and neuropsychological screening can be useful means to obtain individualized information about the patient’s cognitive and emotional status and, where necessary, to guide the choice of subsequent in-depth investigations, in order to better tailor rehabilitative intervention.

## Supporting information

S1 Appendix (XLS)
